# Alternative splicing is required for stage differentiation in malaria parasites

**DOI:** 10.1186/s13059-019-1756-6

**Published:** 2019-08-01

**Authors:** Lee M. Yeoh, Christopher D. Goodman, Vanessa Mollard, Emma McHugh, V. Vern Lee, Angelika Sturm, Anton Cozijnsen, Geoffrey I. McFadden, Stuart A. Ralph

**Affiliations:** 1 Department of Biochemistry and Molecular Biology, Bio21 Molecular Science and Biotechnology Institute, The University of Melbourne, Parkville, 3010 Australia; 2 School of BioSciences, The University of Melbourne, Parkville, 3010 Australia

**Keywords:** *Plasmodium*, *Plasmodium berghei*, Malaria, Splicing, Alternative splicing, SR proteins, Gametocyte, Transcriptome, RNA-seq, Next-generation sequencing

## Abstract

**Background:**

In multicellular organisms, alternative splicing is central to tissue differentiation and identity. Unicellular protists lack multicellular tissue but differentiate into variable cell types during their life cycles. The role of alternative splicing in transitions between cell types and establishing cellular identity is currently unknown in any unicellular organism.

**Results:**

To test whether alternative splicing in unicellular protists plays a role in cellular differentiation, we conduct RNA-seq to compare splicing in female and male sexual stages to asexual intraerythrocytic stages in the rodent malaria parasite *Plasmodium berghei*. We find extensive changes in alternative splicing between stages and a role for alternative splicing in sexual differentiation. Previously, general gametocyte differentiation was shown to be modulated by specific transcription factors. Here, we show that alternative splicing establishes a subsequent layer of regulation, controlling genes relating to consequent sex-specific differentiation of gametocytes.

**Conclusions:**

We demonstrate that alternative splicing is reprogrammed during cellular differentiation of a unicellular protist. Disruption of an alternative splicing factor, *Pb*SR-MG, perturbs sex-specific alternative splicing and decreases the ability of the parasites to differentiate into male gametes and oocysts, thereby reducing transmission between vertebrate and insect hosts. Our results reveal alternative splicing as an integral, stage-specific phenomenon in these protists and as a regulator of cellular differentiation that arose early in eukaryotic evolution.

**Electronic supplementary material:**

The online version of this article (10.1186/s13059-019-1756-6) contains supplementary material, which is available to authorized users.

## Background

Alternative splicing is a phenomenon where different mature transcripts are created from a single pre-mRNA species. In some multicellular eukaryotes, alternative splicing is essential for cellular differentiation to form specific tissues. Alternative splicing generates transcript variants needed to drive cells down a developmental pathway and to maintain the tissue after differentiation has occurred [1, 2]. Indeed, most of the alternative splicing that affects 90% of human genes is tissue-specific [3, 4]. Many unicellular protists undergo stage-specific cellular differentiation, but reported levels of alternative splicing are much lower than in metazoans. For example, the obligate intracellular parasitic eukaryotes of the phylum Apicomplexa [5, 6] have reported splicing rates ranging from 4.5% in *Plasmodium falciparum* [7] to over 20% in *Toxoplasma gondii* [8]. The lower levels of alternative splicing observed may reflect real biological differences, but could also reflect the fact that only a single life stage was used in earlier studies, masking higher levels of alternative splicing that occur during life stage progression.

The transcriptome and proteome of *P. falciparum* are not congruent. Abundance and timing of mRNA appearance correlate poorly with the expression of corresponding proteins [9–11]. This suggests that post-transcriptional regulation processes, perhaps including alternative splicing, are central to apicomplexan biology. Hence, we tested the hypothesis that alternative splicing in unicellular protists plays an important role in life stage differentiation, a phenomenon analogous to tissue-specific splicing in metazoans.

*Plasmodium* species, which cause malaria, are ideal model organisms in which to address this question. These parasites have a complicated life cycle, spanning multiple, morphologically distinct stages in their mammalian and insect hosts. The symptomatic stage in the vertebrate host is the asexual blood cycle, where parasites proliferate rapidly in erythrocytes. Some blood-stage parasites irreversibly convert into sexually dimorphic gametocytes [12]. After ingestion by mosquitoes, female gametocytes activate and egress from their host cell, while male gametocytes produce eight motile gametes (sperm) in a process known as exflagellation [12]. Gametes fuse to form a zygote, which metamorphoses into an ookinete, and subsequently establishes an oocyst in the midgut wall [13]. Sporozoites develop within the oocyst; these migrate to the mosquito salivary glands and are capable of infection of a new vertebrate host [14] via invasion and development of parasites within liver cells in mammalian hosts [15]. The mouse malaria model, *P. berghei*, provides laboratory access to all these life stages, facilitating analysis of the regulation of stage differentiation. In addition, many introns are conserved between *Plasmodium* species, making this an ideal model organism to provide insights into human parasites.

**Fig. 1:**
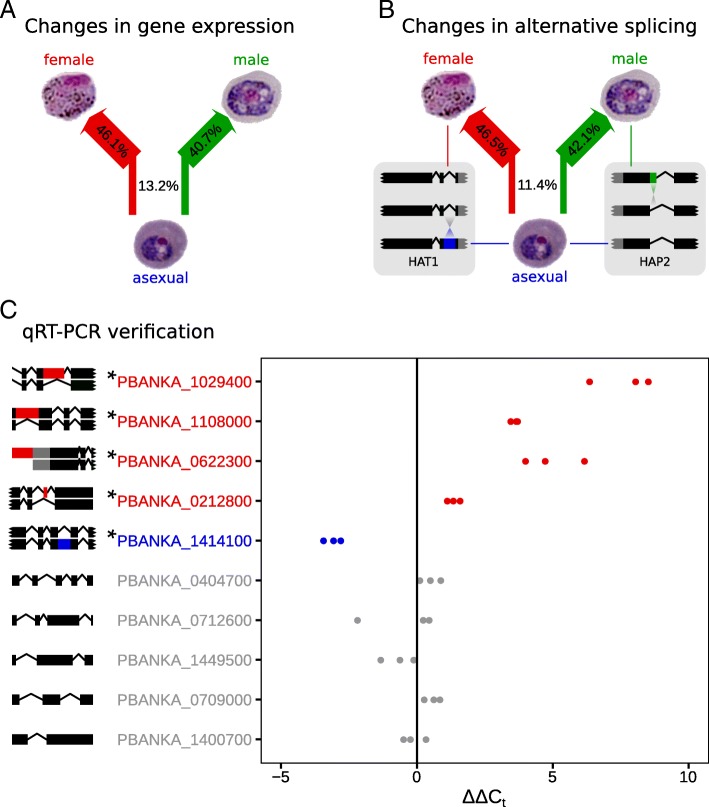
Flowcharts depicting transcriptional changes between stages and qRT-PCR verification. **a** Transcripts are upregulated in both female and male gametocytes or downregulated in both sexes; this comprises 13.1% of genes with detected changes in expression. The remaining genes have changes in their gene expression unique to female and/or male gametocytes. Arrow widths are proportional to the associated percentages, which are derived from the number of genes changing between those stages. (N.B., these do not total 100%, as genes upregulated in females and downregulated in males, or vice versa, are considered “unique” to both sexes). **b** When looking at the changes in alternative splicing, only 11.4% of genes with detected changes in alternative splicing are common to both gametocyte sexes. Two examples of female- and male-specific changes in alternative splicing are depicted. Gene models are to scale, reading sense left-to-right, with the middle model representing the primary isoform. Isoforms linked to specific stages are relatively enriched in that stage, with alternatively spliced regions highlighted. Black rectangles indicate protein-coding regions, gray boxes indicate untranslated regions, and lines indicate introns. **c** RNA-seq analyses revealed changes in alternative splicing between female and asexual parasites in the following genes, which were verified by qRT-PCR. The first four (red) genes were predicted to have a greater proportion of transcripts that retained the highlighted sub-exonic bin in females compared to asexuals (as indicated by *ΔΔC*_*t*_ greater than zero), the next (blue) gene was predicted to have fewer transcripts in females with the highlighted sub-exonic bin (*ΔΔC*_*t*_ less than zero), and the gray genes were predicted to have no changes in splicing (no difference in *ΔΔC*_*t*_). mRNA isoforms to scale are displayed on the left, reading sense left-to-right; sub-exonic bins with a predicted change are highlighted in red or blue. We observed statistical significance in the first five genes, as expected (*p*<0.05, detailed analysis in Additional file 3: Table S3)

Transcriptional reprogramming plays a significant role in the transition from asexual blood stage to gametocytes, with thousands of genes differentially expressed between the stages [16]. 13.1% of all differentially expressed genes in gametocytes were differentially expressed simultaneously in both female and male gametocytes compared to their asexual developmental precursors; this excludes genes that differ when comparing female and male gametocytes directly and, hence, indicates the “common” genes differentially expressed in initial sexual commitment (Fig. 1a, data derived from [16]). Initial differentiation of asexual parasites into gametocytes is triggered by the expression of transcription factors AP2-G and AP2-G2, which regulate whole-gene expression [17]. The importance of these transcription factors in initiating stage transition is emphasized by the increase in asexual growth seen after the ablation of AP2-G [18]. This increase in asexual growth results from a loss of non-replicative gametocytes from the population [17].

## Results and discussion

We used the well-established differential splicing tool, DEXSeq, to examine alternative splicing between these stages [19]. We detected changes in alternative splicing in thousands of genes in both gametocyte sexes compared to their asexual precursors (see Additional file 1: Table S1 and Additional file 2: able S2 for the full list). We detected changes in alternative splicing common to both sexes in 11.4% of genes, again excluding changes between both sexes when compared pairwise (Fig. 1b). Splicing alterations included diverse categories of alternative splicing, including exon skipping, alternative 5^′^ and 3^′^ splice sites, and intron retention. In many cases, the splice changes are predicted to result in truncated or aberrant proteins. These changes in splicing between stages and sexes are viewable for mapped reads preloaded for individual genes using the GBrowse genome browser at www.plasmodb.org [20]. Almost 90% of the changes in alternative splicing events were unique to either male or female gametocytes, suggesting an important role for alternative splicing in sexual determination after the initial commitment to gametocyte development. Indeed, while general gametocyte differentiation is clearly modulated by specific transcriptional factors [17], our analysis indicates that alternative splicing may establish a subsequent layer of regulation, controlling genes relating to consequent sex-specific differentiation of gametocytes.

We attempted to validate changes in alternative splicing by analysis of 15 genes using qRT-PCR. Five samples failed due to poor, non-specific amplification, but ten reactions worked well. In four samples, we predicted higher abundance in a specific sub-exonic bin in female compared to asexual parasites. We predicted lower abundance in one sample, and no change in five control genes. qRT-PCR analysis verified our predictions in all ten cases (Fig. 1c, Additional file 3: Table S3, Additional file 4: Figure S1).

Analysis of sex-specific splicing changes in individual genes reveals changes in several genes that are required for appropriate differentiation (see Additional file 1: Table S1, Additional file 2: Table S2). In female gametocytes, changes in alternative splicing were observed in a major epigenetic regulator histone acetyltransferase HAT1 (PBANKA_0718400) [21], such that retention of the final intron is decreased compared to asexual stages (Fig. 1b). Histone acetyltransferases have been implicated in cell cycle regulation [21], which is consistent with a role in differentiation into female gametocytes.

Differentiation to male gametocytes is accompanied by alternative splicing changes in the male gamete fusion factor HAP2, where we detected relatively more transcripts with a longer first intron in male gametocytes compared to blood stages (PBANKA_1212600, Fig. 1b). In *P. berghei*, HAP2 is essential for fusion of gamete membranes and, hence, fertilisation [22]. HAP2 is upregulated 20-fold in male gametocytes [16], and thus, alternative splicing appears to regulate the function of this gene on an additional level.

**Fig. 2:**
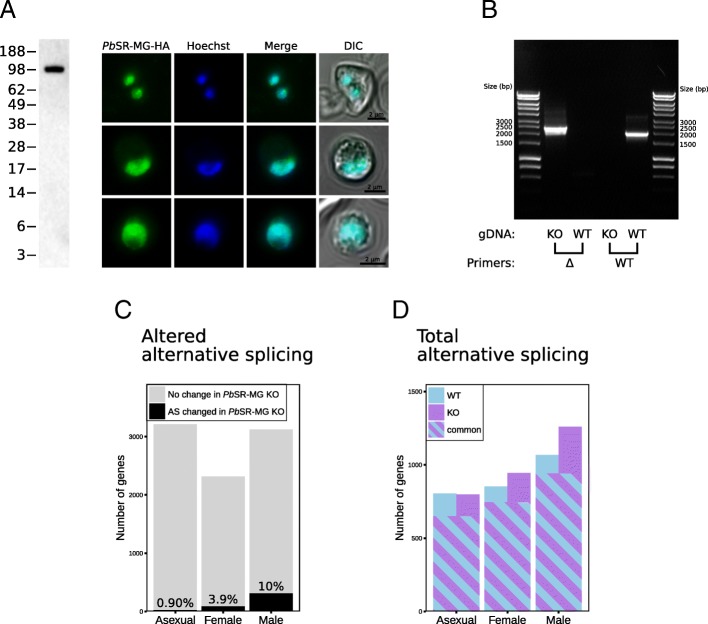
Genetic characterization of *Pb*SR-MG. **a** Western blot and immunofluorescence assay of *Pb*SR-MG-HA transgenic parasites. The western blot was probed with anti-HA, with the predicted mass of the chimeric HA protein at 89.0 kDa. Immunofluorescence assays were captured with confocal microscopy. The green channels shows localization of the HA-tagged *Pb*SR-MG. The blue channels show DNA, which is stained with Hoechst 33342. The merge of these two follows, and the last column adds the brightfield differential interference contrast (DIC) image. **b** PCR screen for *Pb*SR-MG KO vector integration into *P. berghei* ANKA, after monoclonal parasites were selected. **c** Quantification of alternatively spliced genes after ablation of *Pb*SR-MG. After ablation, changes in alternative splicing (AS) can be quantified for each stage. The number of affected genes is low in asexual parasites, moderate in female gametocytes, and highest in males. **d** The total amount of alternative splicing (excluding intron retention) quantified for each stage independently. Total column heights represent total alternatively spliced genes detected in wild type (WT) or *Pb*SR-MG KO (KO). The numbers of genes shared between these samples are indicated by a striped pattern

To explicitly test whether alternative splicing was required to regulate differentiation of gametes, we genetically ablated a putative gametocyte-specific alternative splicing factor, using the PlasmoGEM system, replacing the entire coding sequence of the gene [23, 24]. Serine-arginine–rich (SR) proteins are major regulators of alternative splicing, with regular expression levels necessary for maintaining normal alternative splicing and viability [8, 25]. One SR protein, PBANKA_0921600, is specifically upregulated in male gametocytes of *P. berghei* and downregulated in female gametocytes [16], and phylogenetic analysis identifies this gene as an orthologue of a known splicing factor in *T. gondii* [8]. We named PBANKA_0921600 *Pb*SR-MG (*P*.*berghei* SR protein male gametocytes). Localizing an integrated, hemagglutinin-tagged (HA-tagged) *Pb*SR-MG confirmed its expected nuclear localization (Fig. 2a).

To examine the role of *Pb*SR-MG in regulating splicing, we ablated the gene, obtaining monoclonal parasites (Fig. 2b). We were then able to compare changes in alternative splicing between clonal mutant lines (*Pb*SR-MG KO) and parental lines. RNA-seq analysis revealed differences in alternative splicing to be heavily skewed by sex. Ten percent of genes had modified alternative splicing in male gametocytes, while only 3.9% of female-specific and 0.90% of asexual-stage genes were affected (Fig. 2c, see Additional file 5: Table S4, Additional file 6: Table S5, Additional file 7: Table S6 for full list). Loss of *Pb*SR-MG had minimal impact on transcription, altering the overall expression of only 0.5% of genes (see Additional file 8: Table S7 for full list).

We could now compare *all* male-specific changes in alternative splicing (Fig. 1b, 264+974=1238 genes total) to male genes specifically regulated by *Pb*SR-MG (Fig. 2c, black 10% region, 314 genes total). There were 247 genes that had both male-specific changes in alternative splicing and were male genes regulated by *Pb*SR-MG. Hence, 79% (247/314) of the genes regulated by *Pb*SR-MG were originally identified as having alternative splicing changes specific to male gametocytes. From the opposing perspective, only 20% (247/1238) of all male-specific alternative splicing changes are regulated by *Pb*SR-MG. This contrasts with female gametocytes, where only 5.4% (73/1340) of the female-specific alternative splicing changes are affected by the deletion of *Pb*SR-MG. Presumably, there are other unidentified factors regulating female-specific alternative splicing.

In the analyses presented above, we investigated the changes in alternative splicing between pairs of samples. To assess the total levels of alternative splicing in individual stages, we employed our custom-built JunctionJuror computational tool [8]. This method estimates the abundance of alternative splicing in single samples, although it explicitly underestimates the total amount, because it excludes instances of intron retention. In wild-type parasites, we detected alternative splicing in 800–1100 genes, after the analysis of each sample independently (Fig. 2d, see Additional file 9: Tables S8 for full list). When *Pb*SR-MG was ablated, the number of alternatively spliced genes detected was relatively unchanged for asexual parasites, modestly increased for female gametocytes, and most increased for male gametocytes (Fig. 2d). Many genes were alternatively spliced in both wild-type and mutant parasites (Fig. 2d, striped regions), suggesting that much alternative splicing is regulated by factors additional to *Pb*SR-MG in all three stages.

Ablation of *Pb*SR-MG is associated with increased alternative splicing in males (Fig. 2d, male, WT vs. KO). There are several interpretations consistent with this. *Pb*SR-MG may enhance spliceosomal specificity at alternative splicing sites. When *Pb*SR-MG is absent, the spliceosome acts imprecisely, resulting in aberrant alternative splicing and the production of additional isoforms. Alternatively, *Pb*SR-MG may act as a general suppressor of alternative splicing but this would imply that suppression of alternative splicing is important for male gametocytes. We observed no clear decrease in male alternative splicing compared to the other stages (Fig. 2d, WT, male vs. asexual), so this seems a less compelling mechanism, although it is possible that other splicing factors also regulate alternative splicing in male gametocytes.

Having established changes in alternative splicing as a major phenomemon underlying stage differentiation via the screens above, we wanted to quantify the types of alternative splicing involved. There are presently no integrated computational tools for this kind of analysis, so we applied two different approaches. Unlike our analyses above (Fig. 2c, d), which are agnostic to canonical gene models, these new analyses rely on detecting the differences between observed reads and expected gene models. There are likely to be some differences in genes identified because of these different approaches, but the gene model-based methods do allow us to categorize which types of alternative splicing events occur. We quantified intron retention utilizing featureCounts, which counts the reads that map to defined regions of a gene [26], and quantified alternative 5^′^ and 3^′^ splice site usage and exon skipping using Junction annotation, which allows us to identify junctions that match or conflict with the canonical gene model [27]. We attempted to utilize consistent thresholds with these two methods, so that results between the two software packages were comparable.

**Fig. 3:**
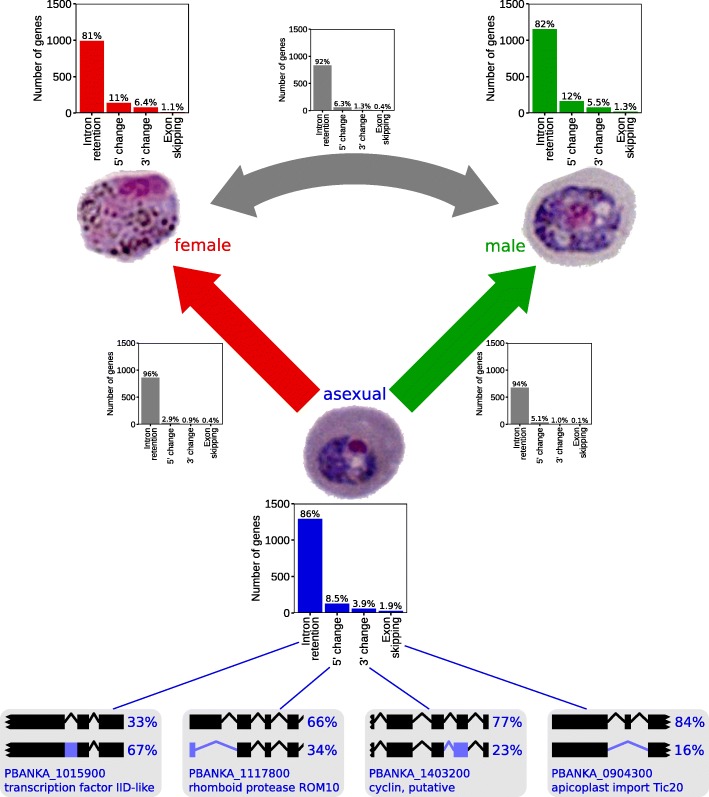
Categorization of alternative splicing in samples. Most alternative splicing observed was intron retention, in both individual stages (coloured bar graphs), and when observing changes in alternative splicing between stages (gray bar graphs). Gene models in the bottom row display representative transcripts for each alternative splicing type. Rectangles indicate protein-coding regions, lines indicate introns, and blue regions indicate non-canonical gene models. The top transcript of each pair represents the canonical gene model from PlasmoDB [20]. The mean proportions are shown next to each isoform

In single stages, intron retention accounted for over 80% of alternatively spliced genes (Fig. 3, colored bar graphs; Additional file 10: Tables S9). Similarly, when looking at alternative splicing that changed between stages, there was also a strong over-representation of intron retention (Fig. 3, gray bar graphs; Additional file 11: Tables S10). In all cases, alternative 5^′^ and 3^′^ splice site usage occured in far fewer genes, and exon skipping contributed a very minor proportion. This distribution of alternative splice types is consistent with the results in plants and fungi [28, 29], where intron retention is the dominant form of alternative splicing, and differs from animals, where intron retention is relatively rare [28], although these analyses did not correlate the potential effect of relative intron size with proportion of intron retention, which might be a confounding factor.

These data were consistent with the broader screen for changes in alternative splicing presented earlier (Additional file 1: Table S1, Additional file 2: Table S2). In the transition from asexual to females, 87% (761/875) of the genes identified in our pipeline were also identified in the screen above. In males, we observed 83% (578/699) overlap.

We also compared wild-type and *Pb*SR-MG KO parasites to analyze the changes in different categories of alternative splicing. We identified only a single gene in both asexual parasites (5^′^ splice site change) and female gametocytes (exon skipping), but observed 60 genes in male gametocytes, with 78% of these identified as undergoing intron retention. These proportions are consistent with the DEXSeq analysis (Fig. 2c), although numbers are smaller and likely reflect the ability of DEXSeq to detect alternative splicing changes that do not correspond to canonical gene models. The gene model-based methods additionally incorporate a fold change threshold for splicing changes, and this also reduces the number of splice changes that are flagged. Hence, the DEXSeq analyses are more likely to capture lower fold change variations, such as those observed in the *Pb*SR-MG KO female parasites.

**Fig. 4:**
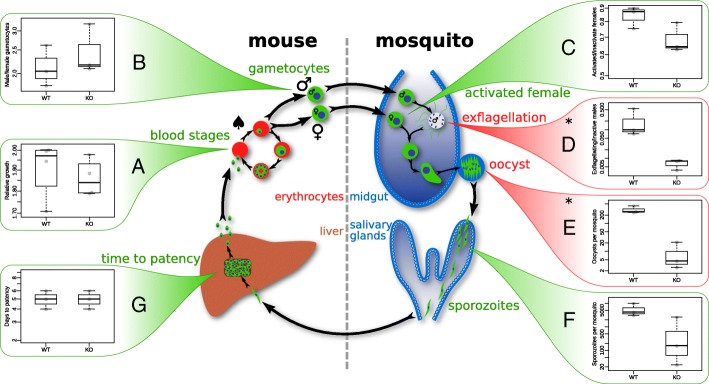
Viability assays through the life cycle of *P. berghei*. We sequenced female gametocytes (♀), male gametocytes (♂), and asexual blood stages (♠). Graphs show comparisons of wild-type (WT) and *Pb*SR-MG KO (KO) parasites at each stage. Growth differences were only observed during male gametocyte exflagellation and oocyst development, marked in red and asterisked (*p < 0.05)

**Fig. 5:**
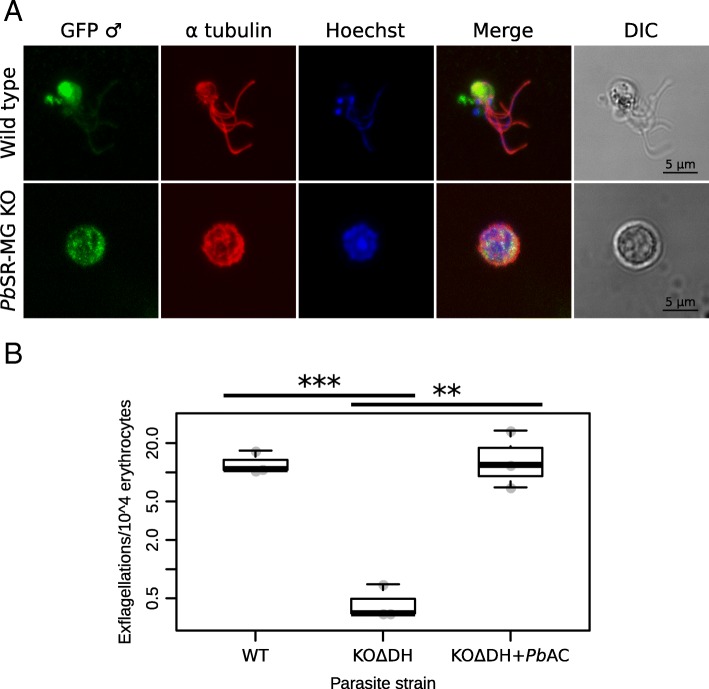
Exflagellation is inhibited in *Pb*SR-MG KO parasites and restored in complemented parasites. **a** Immunofluorescence assay of male gametocytes induced into exflagellation. Green represents a male gametocyte-specific GFP used for sorting. Red and blue indicate alpha-tubulin and DNA, respectively. Wild-type parasites often exhibited conventional exflagellation, as shown in the top row. Very few *Pb*SR-MG KO parasites were able to exflagellate (Fig. 4d), as represented in the bottom row. **b** Complementation of *Pb*SR-MG KO parasites with full-length *Pb*SR-MG on artificial chromosome *Pb*AC0281c08. Exflagellation assay comparing WT, KO *Δ*DH, and KO *Δ*DH+*Pb*AC (*p* value < *0.05; **0.01, ***0.001, ****0.0001)

To determine the biological consequences of changes to alternative splicing caused by the loss of *Pb*SR-MG, we compared the viability of wild-type and *Pb*SR-MG KO parasites through the entire life cycle (Fig. 4). Deletion of *Pb*SR-MG has no detectable effect in asexual blood stages (Fig. 4a, [18]) nor in the proportion of female to male gametocytes produced (Fig. 4b). The proportion of female gametocytes that can be activated is also unchanged (Fig. 4c), but the exflagellation of male gametocytes is severely impacted in *Pb*SR-MG KO parasites (Fig. 4d). When wild-type parasites exflagellate, flagella from the nascent gametes protrude conspicuously from the remnant gametocyte body (Fig. 5a). This occurred in up to 36% of wild-type males after in vitro induction (Fig. 4d). In comparison, we observed almost no exflagellation in *Pb*SR-MG KO parasites (Fig. 4d). Flagella stained with an anti-tubulin antibody remain curled within the gametocyte body (Fig. 5a). Further, on the rare occasion when exflagellation of *Pb*SR-MG KO male gametocytes could be observed, the motility of gametes was markedly impaired, almost to the point of immobility. A similar defect in exflagellation was reported in an SR protein kinase and is consistent with the requirement for phosphorylation to activate *Pb*SR-MG proteins [30].

Ablation of *Pb*SR-MG also resulted in fewer oocysts per mosquito host (Fig. 4e). To test if this difference solely reflected the defect in exflagellation, we repeated the mosquito infection using equal numbers of in vitro generated ookinetes. Despite exposing mosquitoes to equal infective load, we still observed a significant difference in oocyst numbers in *Pb*SR-MG KO compared to wild-type parental parasites (Additional file 4: Figure S2).

No further defects were observed during sporozoite development or in the ability to infect a naive mouse (Fig. 4f, g). Despite the defect in exflagellation, parasites were still able to progress past this stage and complete their life cycle. The *Pb*SR-MG exflagellation phenotype was consistent across clones from independent transfections into three different parasite lines and could be fully complemented by the introduction of full-length *Pb*SR-MG on an artificial chromosome (Fig. 5b, Additional file 4: Figure S3). The oocyst growth defect could also be similarly complemented (Additional file 4: Figure S3).

*Pb*SR-MG clearly plays an important role in stage differentiation, with the development of functional male gametes being significantly impaired by the loss of this splicing factor. There is also a second, less severe effect on the transition from ookinete to oocyst (Fig. 4e). This finding clearly links the changes in alternative splicing resulting from the loss of *Pb*SR-MG with stage-specific development of male gametocytes.

## Conclusions

Initial differentiation of asexual parasites into gametocytes is triggered by expression of transcription factors such as AP2-G and AP2-G2, which regulate whole-gene expression [17]. *Pb*SR-MG appears to be responsible for subsequent differentiation into specific sexes, via regulation of alternative splicing. This multi-stage regulation is further supported by asexual blood stage growth being unaffected by ablation of *Pb*SR-MG, unlike AP2-G ([18], Fig. 4a). This provides additional evidence that *Pb*SR-MG has no role in general gametocytogenesis, instead functioning downstream of this initial commitment.

The central role of post-transcriptional regulation in stage progression has previously been observed in female gametocytes, which regulate gene expression through protein complexes (DOZI and CITH) that stabilize transcripts and temporarily silence their translation [31]. We demonstrate that post-transcriptional regulation by alternative splicing is key to the sexual differentiation of female and male gametes and that a specific alternative splicing factor is essential for the production of viable male gametocytes. Thus, analogous to the regulation of tissue specificity in metazoans, alternative splicing is integral to stage differentiation in these unicellular protists. This reveals alternative splicing as a common mechanism for regulating cellular differentiation in evolutionarily diverse eukaryotes.

## Methods

### Generation of knockout mutants

The knockout vector *Pb*GEM-240478 was obtained from PlasmoGEM [23, 24]. This vector deletes the entire coding sequence for *Pb*SR-MG PBANKA_0921600.

Vectors were transfected into parasites and transfectant parasites selected as previously described [32]. Male Swiss Webster mice aged 4 to 6 weeks old were used in all experiments. Three independent transfections into three different parental parasite lines were performed. The first was into *P. berghei* ANKA wild-type parasites. The second was into *P. berghei* ANKA 820cl1m1cl1 parasites, which produce RFP and GFP in female and male gametocytes, respectively (a kind gift from Andy Waters, University of Glasgow, Glasgow, Scotland) [33]. The final transfection was into *P. berghei* ANKA parasites constitutively expressing tdTomato [34].

After recovery of pyrimethamine-resistant parasites, correct integration of the cassette into the *Pb*SR-MG locus was verified by PCR with the antisense primer “*Pb*SR-MG ASP” and either sense primer “*Pb*GEM GW2+ SP” or “*Pb*SR-MG WT SP” (primer sequences in Additional file 12: Table S11). We generated monoclonal lines via limiting dilution in ten mice per mutant (see Fig. 2b and Additional file 4: Figure S4 for PCR results).

Female and male gametocyte mutants based on the 820cl1m1cl1 line [33], as well as asexual erythrocytic stages, were purified based on differential density and on fluorescent sorting and sequenced as previously reported [16].

### Generation of epitope-tagged mutant

The hemagglutinin epitope-tagged line was created by modification of a pL0006-based plasmid [35]. This plasmid was designed for single-site recombination into *P. berghei* and includes three hemagglutinin (HA) tags, followed by the 3^′^-UTR of KAHRP, followed by a DHFR selectable marker. We cloned the 3^′^ end of *Pb*SR-MG from genomic DNA with the primers “*Pb*SR-MG 3^′^-HindIII-ApaI SP” and “*Pb*SR-MG 3^′^-AvrII-BglII ASP” (primer sequences in Additional file 12: Table S11) and ligated this into the vector backbone.

The integrity of the plasmid was confirmed by sequencing. The final vector was linearized with the restriction enzyme *Bsr*GI-HF, and *P. berghei* ANKA wild-type parasites transfected as above.

Parasites were screened with the sense primer “*Pb*SR-MG test SP” and either antisense primer “*Pb*SR-MG 3^′^ UTR test ASP” or “p*Pb*3HA 3’ UTR ASP” (primer sequences in Additional file 12: Table S11). The strongest signal in transfected parasites was for the mutant locus (see Additional file 4: Figure S4).

The epitope was visualized in blood-stage parasites via western blot. Samples were run on CF11 columns to deplete leukocytes, before being resuspended in 0.15% *w*/*v* saponin in Dulbecco’s phosphate-buffered saline and incubated for 10 min. Samples were washed four times, before resuspension in sample buffer and running on a NuPage SDS-PAGE gel (Invitrogen, Australia). Blots were probed with rat anti-HA (1/200; Roche, Australia, 18674) and anti-rat horseradish peroxidase (1/1000; Pierce, Australia, 31470). The apparent mass of 98 kDa was close to the predicted mass of 89.0 kDa.

### Immunofluorescence assays

Immunofluorescence assays of HA-tagged parasites were performed on asexual blood stages, and assays were also performed on exflagellating male gametocytes, comparing knockout and parental 820cl1m1cl1 parasites, as previously described [36, 37], with the following differences.

For blood-stage assays, samples were fixed in 4% paraformaldehyde and 0.00625% glutaraldehyde. For exflagellating gametocytes, 4% paraformaldehyde and microtubule stabilization buffer [37] were used. Parasites were permeabilied for 10 min in 0.1% or 0.5% Triton X-100. Blood-stage parasites were incubated in rat anti-HA (1/250; Roche, Australia, 18674) and Alexa Fluor ^*Ⓡ*^ goat anti-rat-488 (1/5000; Life Technologies, Australia, A-11006). Exflagellating gametocytes were incubated in chicken anti-GFP (1/1000; Abcam, USA, ab101863) and mouse anti-alpha-tubulin (1/300; Sigma-Aldrich, Australia, T9026), followed by Alexa Fluor ^*Ⓡ*^ goat anti-chicken-488 (1/2000; Life Technologies, Australia, A-11039) and Alexa Fluor ^*Ⓡ*^ goat anti-mouse-546 (1/1000; Life Technologies, Australia, A-11003). Both stages were incubated in 200 µg/ml Hoechst 33342 (Life Technologies, Australia).

Images were acquired with a Leica SP2 confocal microscope, linearly adjusted for brightness and contrast, and merged using the bundled Leica software.

### Bioinformatic analyses

Wild-type female, male, and asexual transcriptomes were previously published in [16]. RNA-seq mapping and differential gene expression analyses were performed as previously described [16]. Data verification and quality control were performed as previously described [16] and included generation of multidimensional scaling plots and heatmaps (Additional file 4: Figure S5–S6). Alternative splicing analyses were performed as previously described [8], using DEXSeq for changes in splicing between the two samples [19], and JunctionJuror for the presence of alternative splicing in single samples [8]. Full commands and version numbers are presented in Additional file 13: Text 1.

Categorization of alternative splicing type (Fig. 3) was achieved using two tools. Intron retention was quantified with featureCounts [26] and alternative 5^′^/ 3^′^ splicing and exon skipping using Junction annotation, in the RSeQC package [27]. For both approaches, we set a minimum threshold of three reads per replicate for the alternative junction to be accepted for further analysis. The intron read counts were normalized for intron length. We filtered for alternative splicing events where the alternative junction reads were more than 10% of the total splicing observed for each junction.

To analyze splicing changes between stages, we compared the fractions of alternative splicing reads for each junction, and obtained corrected *p* values using Benjamini-Hochberg false discovery correction, filtering at 0.25 FDR. We again filtered for junctions with a minimum coverage of three reads and changes that exceeded 10% of the total transcripts. We also filtered out differential alternative splicing events where the magnitude of the relative change was less than twofold in abundance. Because differential expression cannot be distinguished from differential splicing when the gene has negligible expression in one condition, we also excluded junctions with an average of fewer than 10 canonical junction per replicate for any condition.

### qRT-PCR verification

Prior to reverse transcription, RNA samples were treated with DNase I (Qiagen, Australia). This comprized one asexual wild-type sample and one female wild-type sample. cDNA was synthesized using the SensiFAST^TM^ cDNA Synthesis Kit (Bioline, Australia) according to the manufacturer’s instructions. Quantitative PCR was performed with the SensiFAST^TM^ SYBR ^*Ⓡ*^ Hi-ROX Kit on an Applied Biosystems Quant Studio 6 Flex (Life Technologies, Australia). This was repeated three times for each sample. The *ΔΔC*_*t*_ for the average of three experiments was calculated with *ΔΔC*_*t*_=*ΔC*_*t*_(asexual)−*ΔC*_*t*_(female), where *ΔC*_*t*_ compared two amplicons from the same gene, with the numerator as the amplicon associated with the exonic region with changed expression (the “_T” primer), and the denominator representing the amplicon for an unchanged region (the “_C” primer). Statistical analysis was performed with Welch’s one-sample *t* test. Primer sequences are listed in Additional file 12: Table S11.

### Growth assays

Blood stage growth assays were performed by competition of tdTomato parasites with parasites expressing GFP constitutively under the control of the HSP70 promoter (a kind gift from Robert Ménard; promoter described in [38]). Naive mice were infected with 5×10^4^ parasites of either *Pb*SR-MG KO or parental tdTomato strains, plus 5×10^4^ parasites of the GFP strain, each combination with four replicates. Parasites were counted by FACS 3–6 days post-infection. For each mouse and each day, the parasitemia of tdTomato strains was normalized to that of the GFP strain. We presented this visually by dividing this normalized growth at 6 days by that at 3 days (Fig. 4). For statistical tests, normalized *Pb*SR-MG KO tdTomato parasites were divided by normalized parental tdTomato parasites for each day. To see if this ratio changed as parasitemia increased, we assayed by simple linear regression, with no change in ratio over time detected (*p* value = 0.886).

Gametocytes ratios were determined by comparison of female and male counts of 820cl1m1cl1-based parasites, as partitioned by FACS sorting. See [16] for a representative FACS plot.

Activated females were quantified as previously described [39]; the numbers reported are the sum of activated females and ookinetes, since the latter are activated females that have been subsequently fertilized. This was performed in tdTomato-based parasites.

The exflagellation rates presented were determined from 820cl1m1cl1-based parasites. In vitro induction of exflagellation was performed as previously described [39], but using 2.5 µl blood in 50 µl exflagellation media. After 13 min, parasites were fixed as described above for immunofluorescence assays. Males were detected by the presence of the male-specific GFP marker, and exflagellating males were identified by the protrusion of tubulin (Fig. 5a). Exflagellation defects were confirmed in all three knockout lines.

Mosquitoes were infected as previously described [40]. Given the low number of exflagellation events in *Pb*SR-MG KO mutants, we were sometimes forced to infect mosquitoes with fewer than the normal minimum limit of exflagellating gametocytes [40].

Oocyst and sporozoite numbers were quantified by analysis of tdTomato parental and *Pb*SR-MG KO parasites in paired experiments, as previously described [40], but without staining. Time to patency in mice reinfected with these parasites was determined as previously described [40]. To control for lack of growth in upstream mosquito parasite stages, mice that exhibited no infection after several weeks were not included in the analysis.

Normalized oocyst growth data (Additional file 4: Figure S2) were obtained by in vitro fertilization of blood-stage parasites (using either 820cl1m1cl1 or tdTomato lines) as previously reported [39]. Parasites were diluted to 460 or 785 ookinetes per microliter blood and fed to mosquitoes via an artificial membrane. Due to the low numbers of ookinetes acquired in the *Pb*SR-MG KO strain, the exact number of normalized ookinetes varied between experiments. Hence, KO and parental strains were experimentally paired and subsequently analyzed with a paired *t* test.

Complementation was achieved by growing 820cl1m1cl1-based *Pb*SR-MG KO parasites in mice treated with 1 mg/ml 5-fluorocytosine for 4 days, to select for parasites that had lost the DHFR-yFCU cassette due to recombination (Additional file 4: Figure S3A). Parasites without the hDHFR-yFCU cassette were cloned by limiting the dilution, and the recovery of a clone lacking hDHFR-yFCU (*Pb*SR-MG-KO *Δ*DH) was confirmed by PCR (Additional file 4: Figure S3B). The PlasmoGEM construct containing the full-length *Pb*SR-MG gene on artificial chromosome *Pb*AC0281c08 was linearized with *Not*I, transfected into *Pb*SR-MG-KO *Δ*DH parasites, and transfectants selected by treatment with pyrimethamine. The genotype of the complemented parasite line was confirmed by PCR (Additional file 4: Figure S3B). Exflagellation counts were obtained as previously described [41]. Oocyst counts were obtained as above.

### Statistical analysis

For all graphs in Figs. 4 and 5b and Additional file 4: Figure S3, boxplots and medians are based on log-transformed data. Whiskers are restricted to 1.5 times the interquartile range, in log-space. With the exception of the blood stage competition assay, which is described above, statistical significance was inferred using Welch’s two-sample *t* test on log-transformed data. Counts that were zero (e.g., exflagellation of *Pb*SR-MG KO parasites) were converted to the limit of detection (i.e., 0.5 parasites per sample) for the purposes of these calculations. *p* values of < 0.05 were considered statistically significant for all tests. All statistical tests were two-sided.

## Additional files

Additional file 1 Table S1. A tab-delimited text file containing a list of genes where alternative splicing has changed in female gametocytes compared to asexual erythrocytic stages (in wild-type parasites). (TSV 527 kb)

Additional file 2 Table S2. A tab-delimited text file containing a list of genes where alternative splicing has changed in male gametocytes compared to asexual erythrocytic stages (in wild-type parasites). (TSV 487 kb)

Additional file 3 Table S3. A tab-delimited text file containing qRT-PCR results that verify changes in alternative splicing of ten genes identified by RNA-seq data in Additional file 1: Table S1. (TSV 2 kb)

Additional file 4 Figure S1. A PDF image showing changes in alternative splicing between samples validated by qRT-PCR. Figure S2. A PDF image showing growth of *Pb*SR-MG KO compared to parental parasites after normalization of ookinetes in all samples. Figure S3. A PDF image showing complementation of *Pb*SR-MG KO parasites with full-length *Pb*SR-MG. Figure S4. A PDF image showing PCR screens of monoclonal parasites for *Pb*SR-MG KO integration into *P. berghei* ANKA 820cl1m1cl1 and *P. berghei* ANKA tdTomato; PCR analysis of polyclonal epitope-tagged *Pb*SR-MG 3^′^-HA. Figure S5. A PDF image showing a multidimensional scaling (MDS) plot based on gene-level differential expression. Figure S6. A PDF image showing a heatmap of gene-level differential expression. (PDF 1172 kb)

Additional file 5 Table S4. A tab-delimited text file containing a list of genes where alternative splicing has changed in *Pb*SR-MG KO parasites compared to parental parasites, for asexual erythrocytic stages. (TSV 6 kb)

Additional file 6 Table S5. A tab-delimited text file containing a list of genes where alternative splicing has changed in *Pb*SR-MG KO parasites compared to parental parasites, for female gametocytes. (TSV 20 kb)

Additional file 7 Table S6. A tab-delimited text file containing a list of genes where alternative splicing has changed in *Pb*SR-MG KO parasites compared to parental parasites, for male gametocytes. (TSV 86 kb)

Additional file 8 Table S7. A tab-delimited text file containing a list of genes with differential expression of full genes in *Pb*SR-MG KO parasites compared to parental parasites, for all stages. (TSV 5 kb)

Additional file 9 Tables S8. A zip-compressed file of tab-delimited text files containing six lists of genes that are constitutively alternatively spliced (excluding intron retention), for all samples. (ZIP 15 kb)

Additional file 10 Tables S9. An Excel spreadsheet quantifying categories of alternative splicing in single stages. (XLSX 6008 kb)

Additional file 11 Tables S10. An Excel spreadsheet quantifying categories of alternative splicing between stages. (XLSX 986 kb)

Additional file 12 Table S11. A tab-delimited text file containing a list of primers used in this study. (TSV 3 kb)

Additional file 13 Text 1. A text file containing detailed bioinformatics methods with full commands and version numbers. (TXT 4 kb)
