# Supplementary material for: Alternative splicing is required for stage differentiation in malaria parasites
Source: Genome Biol. 2019 Aug 1;20:151. doi: 10.1186/s13059-019-1756-6 (PMC6669979; doi:10.1186/s13059-019-1756-6)
Supplement: Supplementary file 4 — Figure S1. A PDF image showing changes in alternative splicing between samples validated by qRT-PCR. Figure S2. A PDF image showing growth of PbSR-MG KO compared to parental parasites after normalization of ookinetes in all samples. Figure S3. A PDF image showing complementation of PbSR-MG KO parasites with full-length PbSR-MG. Figure S4. A PDF image showing PCR screens of monoclonal parasites for PbSR-MG KO integration into P. berghei ANKA 820cl1m1cl1 and P. berghei ANKA tdTomato; PCR analysis of polyclonal epitope-tagged PbSR-MG 3′-HA. Figure S5. A PDF image showing a multidimensional scaling (MDS) plot based on gene-level differential expression. Figure S6. A PDF image showing a heatmap of gene-level differential expression. (PDF 1172 kb) [file 13059_2019_1756_MOESM4_ESM.pdf]

Figure S1

Changes in alternative splicing as identified by RNA-seq data, for PBANKA_1029400, PBANKA_1108000, PBANKA_0622300, PBANKA_0212800, and PBANKA_1414100 respectively. Biological replacates are shown for wild-type female and wild-type asexual parasites. These were validated by qRT-PCR, with primers for the region with changes in expression marked on the lower two tracks.

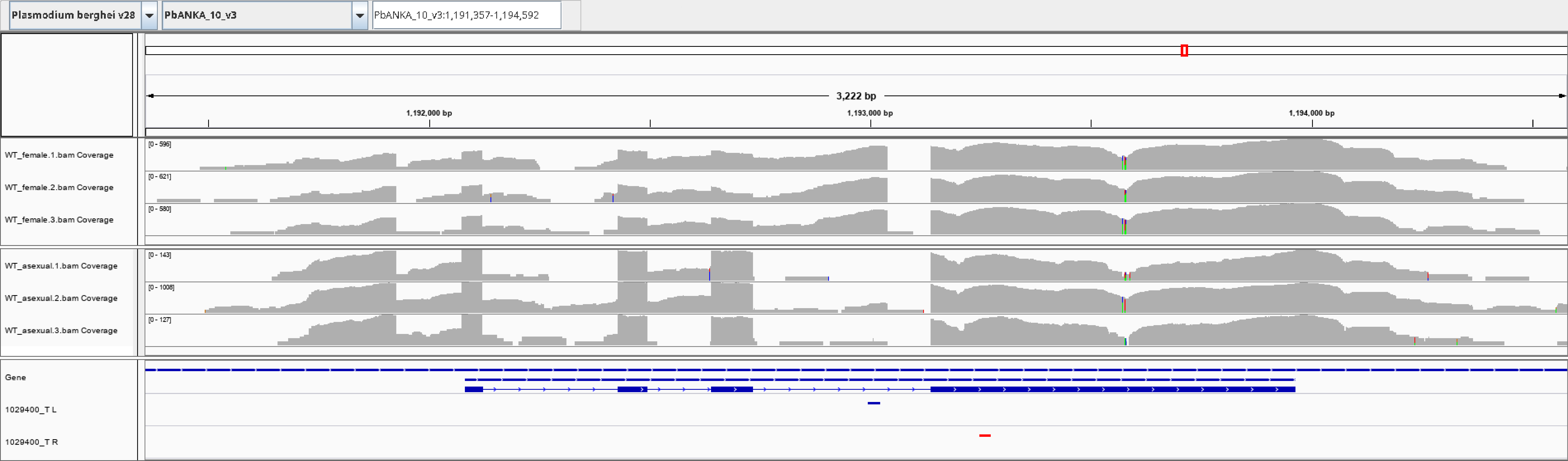

Plasmodium berghei v28
PbANKA_10_v3
PbANKA_10_v3:1,191,357-1,194,592
3,222 bp
1,192,000 bp
1,193,000 bp
1,194,000 bp
WT_female.1.bam Coverage
[0 - 596]
WT_female.2.bam Coverage
[0 - 621]
WT_female.3.bam Coverage
[0 - 580]
WT_asexual.1.bam Coverage
[0 - 143]
WT_asexual.2.bam Coverage
[0 - 1008]
WT_asexual.3.bam Coverage
[0 - 127]
Gene
1029400_T L
1029400_T R

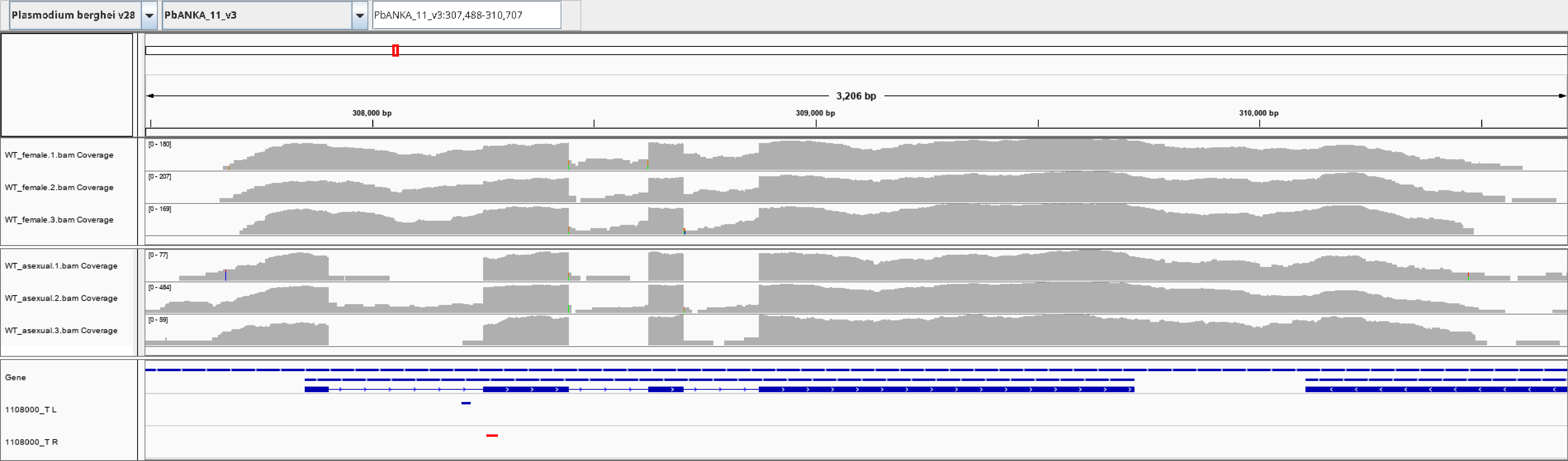

Plasmodium berghei v28
PbANKA_11_v3
PbANKA_11_v3:307,488-310,707
3,206 bp
308,000 bp
309,000 bp
310,000 bp
WT_female.1.bam Coverage
[0 - 180]
WT_female.2.bam Coverage
[0 - 207]
WT_female.3.bam Coverage
[0 - 169]
WT_asexual.1.bam Coverage
[0 - 77]
WT_asexual.2.bam Coverage
[0 - 484]
WT_asexual.3.bam Coverage
[0 - 59]
Gene
1108000_T L
1108000_T R

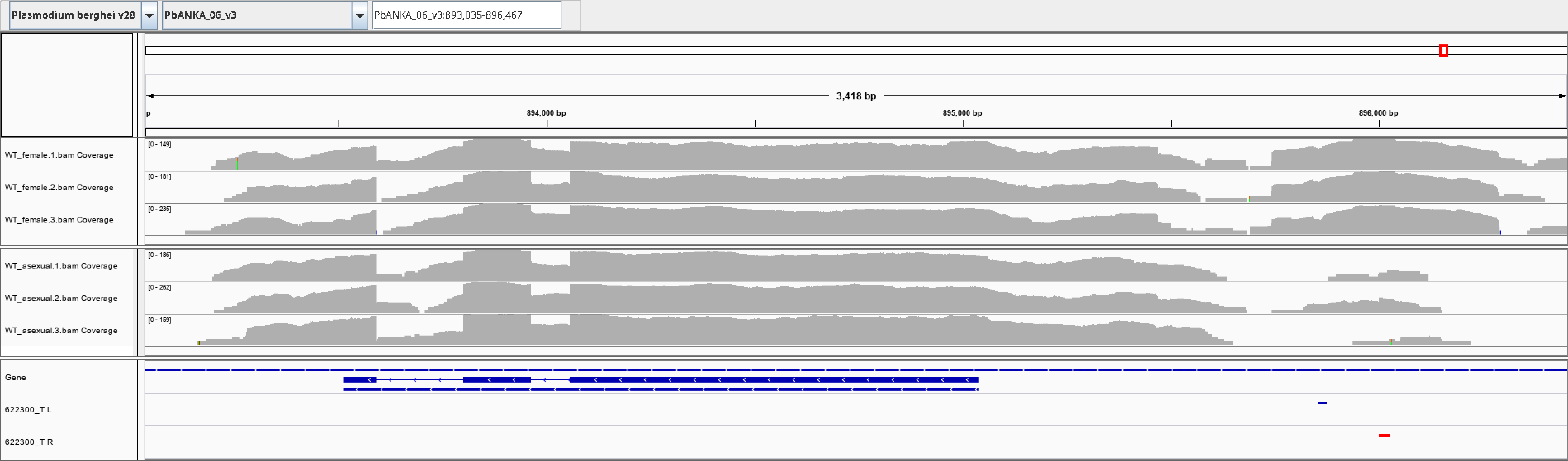
Plasmodium berghei v28
PbANKA_06_v3
PbANKA_06_v3:893,035-896,467
3,418 bp
894,000 bp
895,000 bp
896,000 bp
WT_female.1.bam Coverage
[0 - 149]
WT_female.2.bam Coverage
[0 - 181]
WT_female.3.bam Coverage
[0 - 235]
WT_asexual.1.bam Coverage
[0 - 186]
WT_asexual.2.bam Coverage
[0 - 262]
WT_asexual.3.bam Coverage
[0 - 159]
Gene
622300_T L
622300_T R

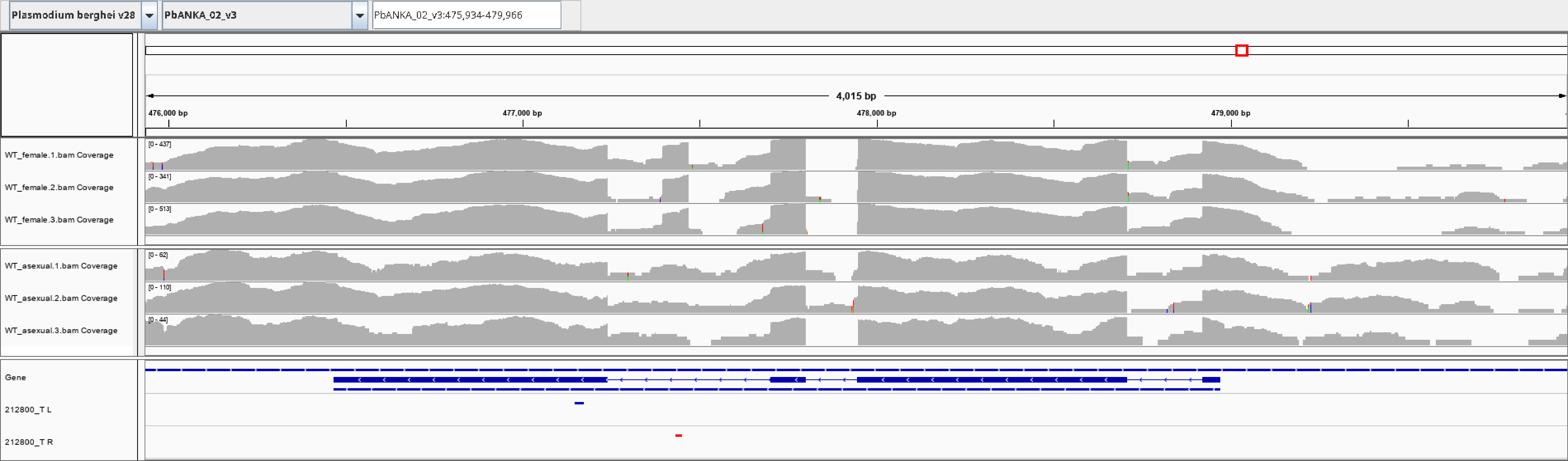
Plasmodium berghei v28
PbANKA_02_v3
PbANKA_02_v3:475,934-479,966
4,015 bp
476,000 bp
477,000 bp
478,000 bp
479,000 bp
WT_female.1.bam Coverage
[0 - 437]
WT_female.2.bam Coverage
[0 - 341]
WT_female.3.bam Coverage
[0 - 513]
WT_asexual.1.bam Coverage
[0 - 62]
WT_asexual.2.bam Coverage
[0 - 110]
WT_asexual.3.bam Coverage
[0 - 44]
Gene
212800_T L
212800_T R

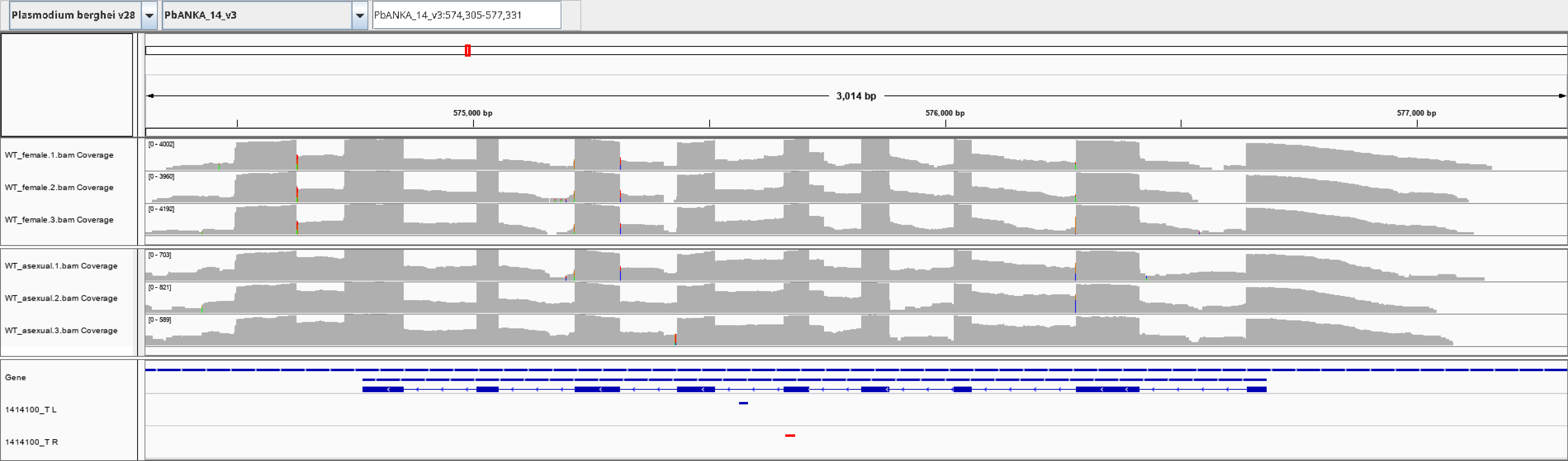
Plasmodium berghei v28
PbANKA_14_v3
PbANKA_14_v3:574,305-577,331
3,014 bp
575,000 bp
576,000 bp
577,000 bp
WT_female.1.bam Coverage
[0 - 4002]
WT_female.2.bam Coverage
[0 - 3960]
WT_female.3.bam Coverage
[0 - 4192]
WT_asexual.1.bam Coverage
[0 - 703]
WT_asexual.2.bam Coverage
[0 - 821]
WT_asexual.3.bam Coverage
[0 - 589]
Gene
1414100_T L
1414100_T R

Figure S2

Growth of wild-type and *Pb*SR-MG KO oocysts after normalisation of ookinetes. The graph compares wildtype (WT) and *Pb*SR-MG KO (KO) parasites. Statistically significant differences in growth were observed (p value = 0.026).

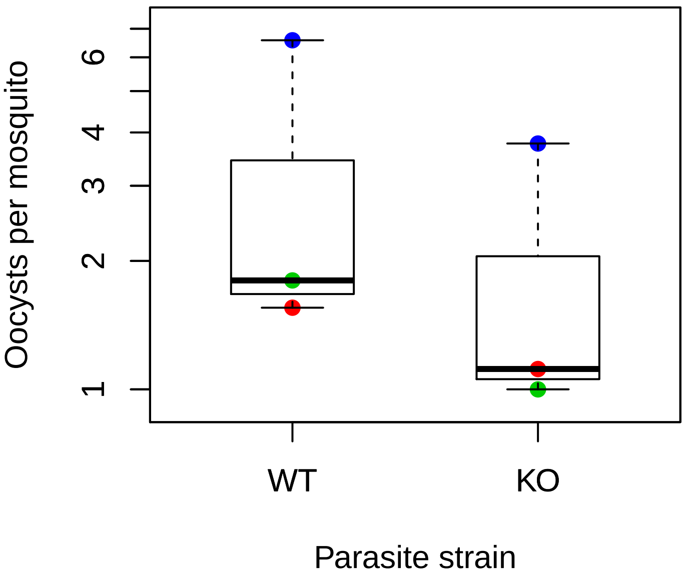

Oocysts per mosquito
1
2
3
4
6
WT
KO
Parasite strain

Figure S3

Complementation of *Pb*SR-MG KO parasites with full-length *Pb*SR-MG on artificial chromosome *Pb*AC0281c08. A) Schematic of the constructs used to generate an exogenously complemented *Pb*SR-MG KO i) wild type, genomic locus *Pb*SR-MG (WT) ii) deleted locus with hDHFR-yFCU cassette flanked by homologous sequences (blue squares) (KO) iii) removal of hDHFR-yFCU by recombination in parasites treated with 5-fluorocytosine (KOΔDH) iv) *Pb*AC0281c08 artificial chromosome transfected into KOΔDH (KOΔDH+*Pb*AC). B) PCR genotyping showing primer pairs a) amplifying *Pb*SR-MG in WT genomic context b) amplifying *Pb*SR-MG in either genome or artificial chromosome c) amplifying *Pb*SR-MG KO d) discriminating KO from KOΔDH. C) Oocyst-growth assay comparing WT, KOΔDH and KOΔDH+*Pb*AC (p value < *0.05; **0.01, ***0.001, ****0.0001).

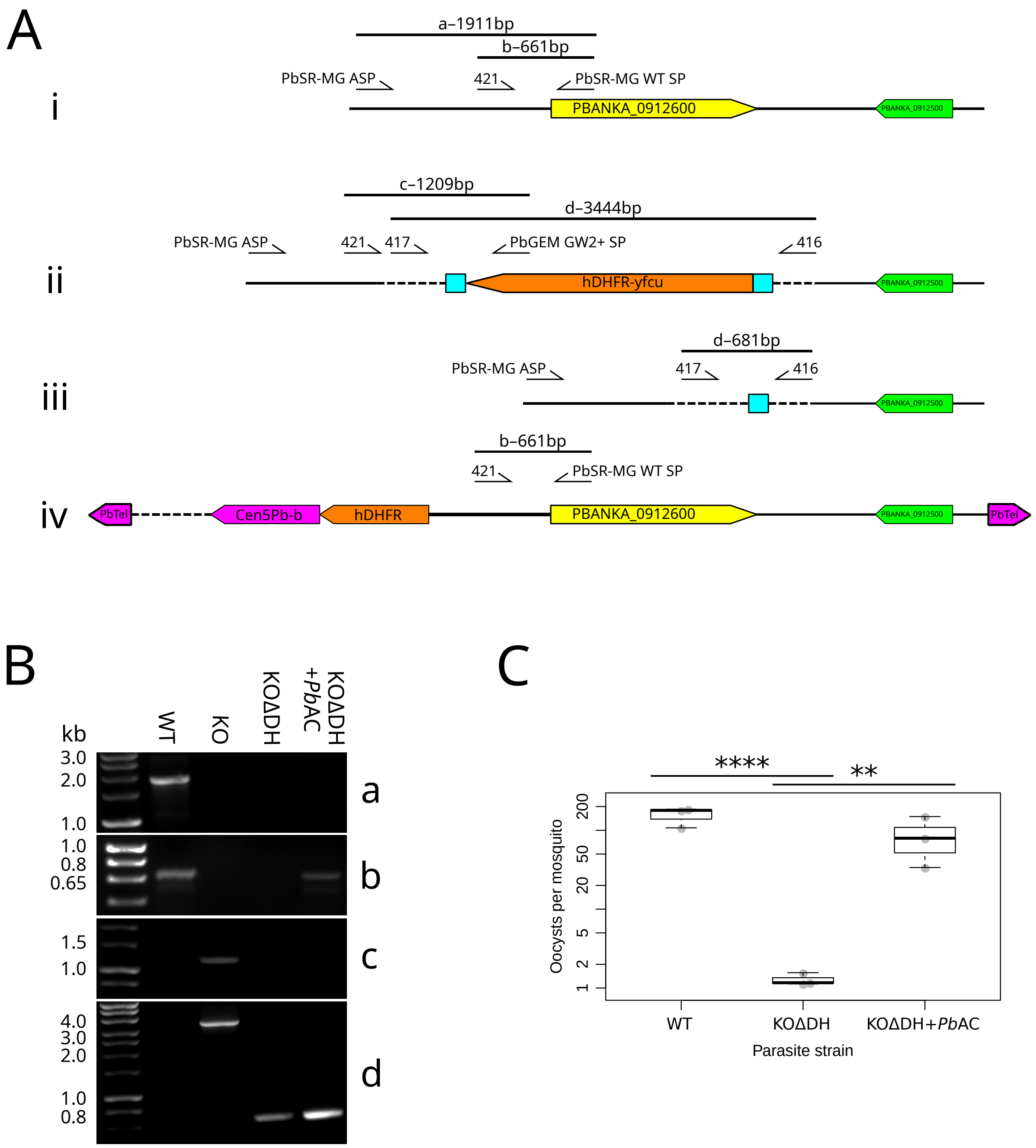
A
i
a–1911bp
b–661bp
PbSR-MG ASP
421
PbSR-MG WT SP
PBANKA_0912600
PBANKA_0912500
ii
c–1209bp
d–3444bp
PbSR-MG ASP
421
417
PbGEM GW2+ SP
416
hDHFR-yfcu
PBANKA_0912500
iii
d–681bp
PbSR-MG ASP
417
416
PBANKA_0912500
iv
b–661bp
421
PbSR-MG WT SP
PbTel
Cen5Pb-b
hDHFR
PBANKA_0912600
PBANKA_0912500
PbTel
B
kb
WT
KO
KOΔDH
KOΔDH +PbAC
3.0
2.0
1.0
a
1.0
0.8
0.65
b
1.5
1.0
c
4.0
3.0
2.0
1.0
0.8
d
C
****
**
Oocysts per mosquito
200
50
20
5
2
1
WT
KOΔDH
KOΔDH+PbAC
Parasite strain

Figure S4

PCR screen for vector integration into *Pb*SR-MG transgenic parasites. The *Pb*SR-MG KO vector was transfected into *P. berghei* ANKA 820cl1m1cl1 and *P. berghei* ANKA tdTomato, with monoclonal parasites screened. Epitope-tagged *Pb*SR-MG 3′-HA was screened for the presence of integration in polyclonal parasites.

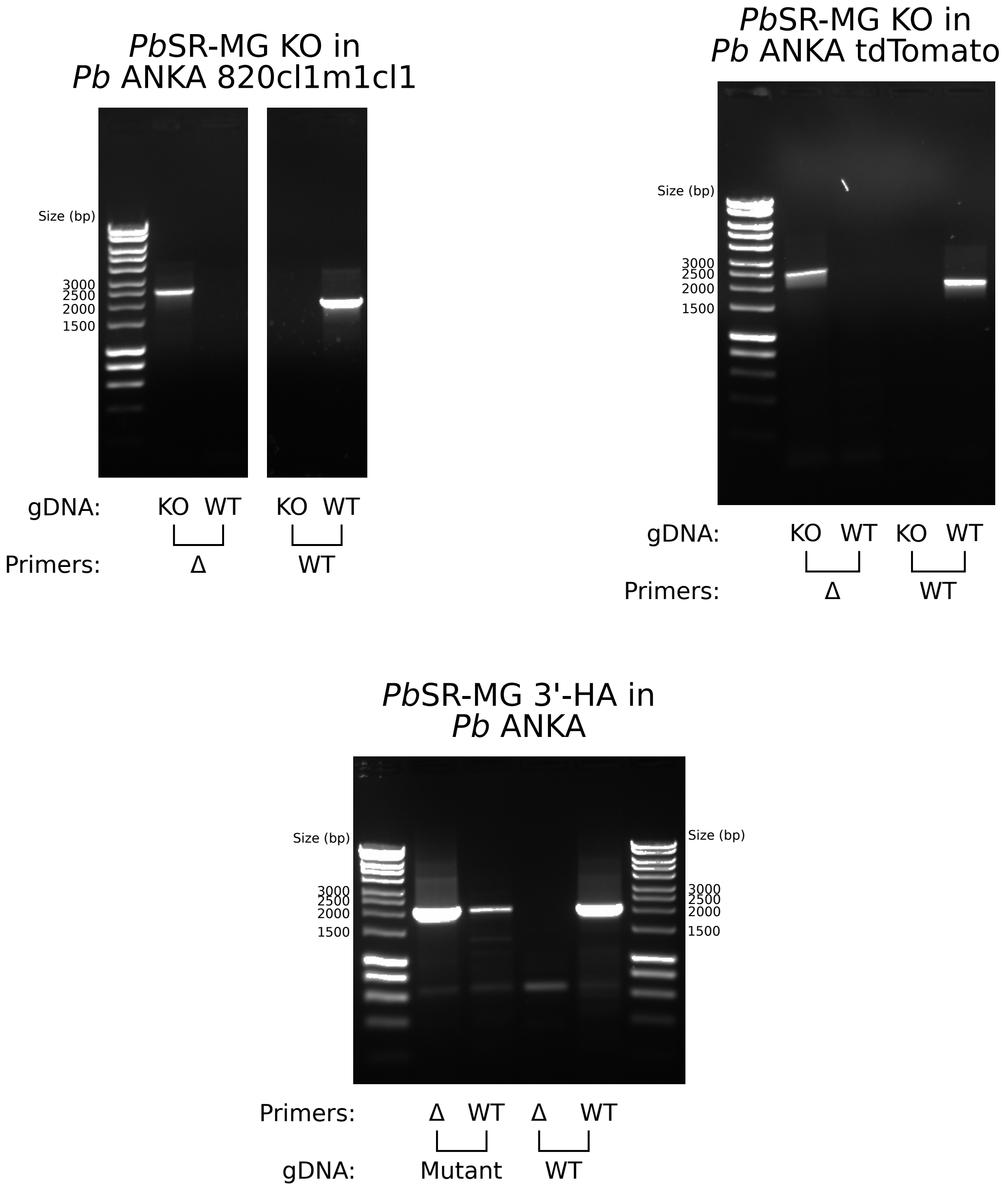
PbSR-MG KO in
Pb ANKA 820cl1m1cl1
Size (bp)
3000
2500
2000
1500
gDNA: KO WT KO WT
Primers: Δ WT
PbSR-MG KO in
Pb ANKA tdTomato
Size (bp)
3000
2500
2000
1500
gDNA: KO WT KO WT
Primers: Δ WT
PbSR-MG 3'-HA in
Pb ANKA
Size (bp)
3000
2500
2000
1500
Size (bp)
3000
2500
2000
1500
Primers: Δ WT Δ WT
gDNA: Mutant WT

Figure S5

Multidimensional scaling (MDS) plot based on gene-level differential expression.

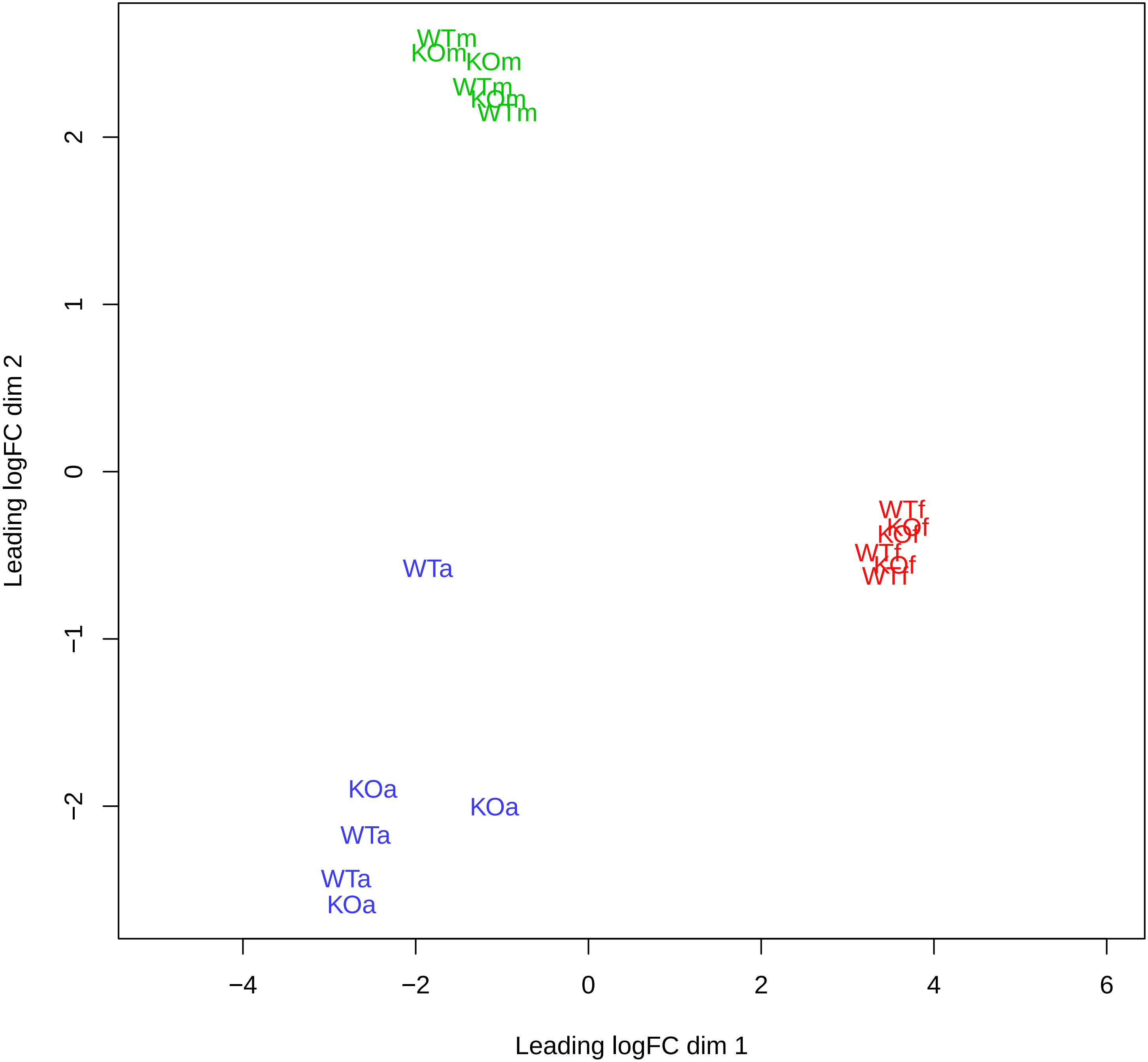
WTm
KOm
KOm
WTm
KOm
WTm
WTf
KOf
KOf
WTf
KOf
WTf
WTa
KOa
KOa
WTa
WTa
KOa
Leading logFC dim 1
Leading logFC dim 2
−4
−2
0
2
4
6
−2
−1
0
1
2

Figure S6

Heatmap of gene-level differential expression showing sample clustering similar to MDS plot. Bars above the heatmap denote stages, with red as female, green as male, and blue as asexual. Labels below the heatmap indicate w for wild-type and k for *Pb*SR-MG KO.

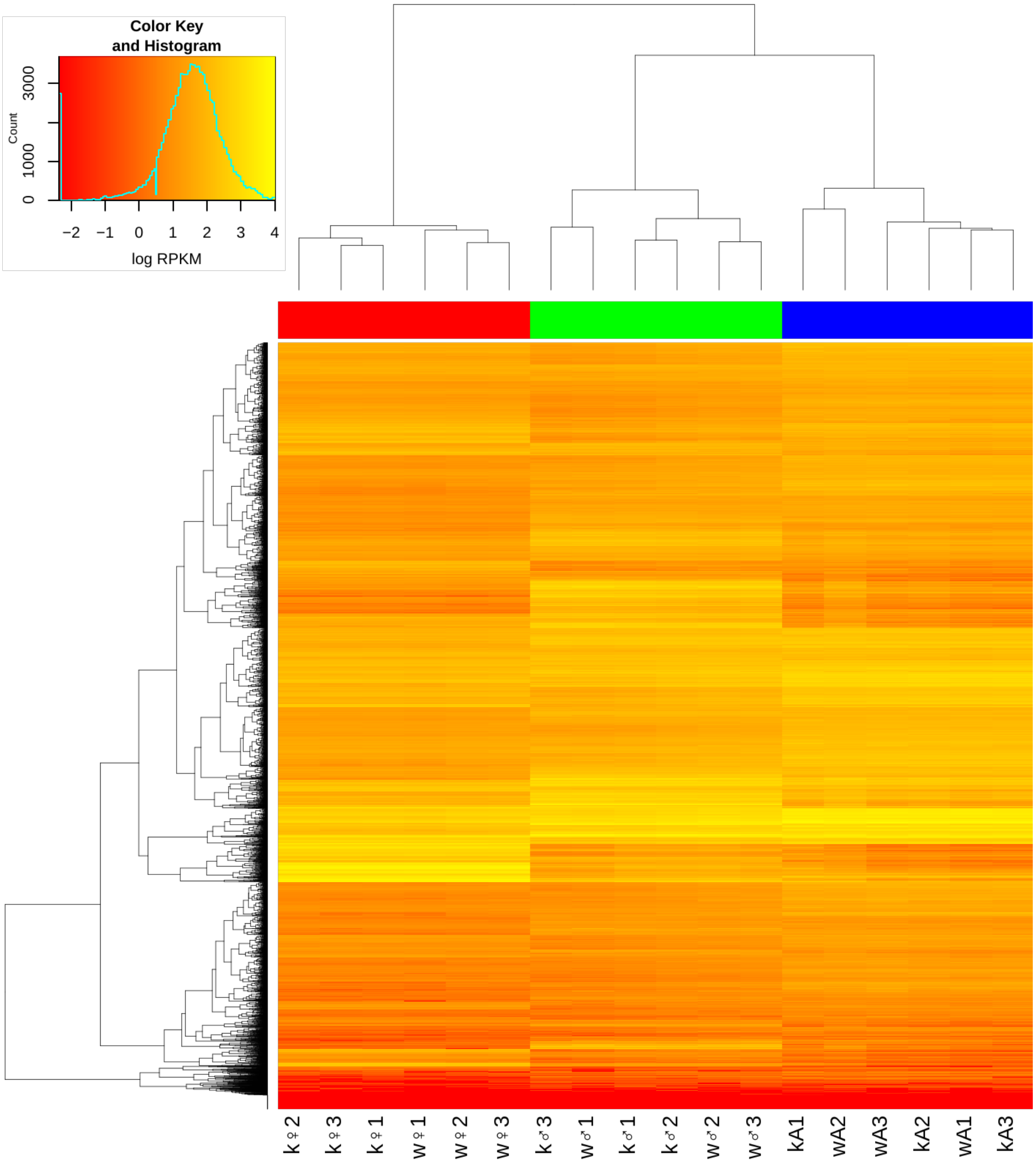
Color Key
and Histogram
Count
0
1000
3000
−2
−1
0
1
2
3
4
log RPKM
k♀2
k♀3
k♀1
w♀1
w♀2
w♀3
k♂3
w♂1
k♂1
k♂2
w♂2
w♂3
kA1
wA2
wA3
kA2
wA1
kA3
